# Knowledge level and health information-seeking behavior of people with diabetes in rural areas: a multicenter cross-sectional study

**DOI:** 10.3389/fpubh.2024.1285114

**Published:** 2024-05-01

**Authors:** Yudong Wang, Yanping Zhang, Tingting Guo, Jiaxia Han, Guifen Fu

**Affiliations:** ^1^Department of Nursing, Liaocheng People’s Hospital, Liaocheng, China; ^2^Department of Geriatric Endocrinology and Metabolism, The People’s Hospital of Guangxi Zhuang Autonomous Region, Nanning, China; ^3^Department of Nursing, The People's Hospital of Guangxi Zhuang Autonomous Region, Nanning, China; ^4^School of Nursing, Youjiang Medical University for Nationalities, Baise, China; ^5^Department of Endocrinology and Metabolism, The People’s Hospital of Guangxi Zhuang Autonomous Region, Nanning, China

**Keywords:** diabetes, complications, prevalence, diabetes knowledge, health information-seeking behavior

## Abstract

**Introduction:**

There is a lack of research on the current level of diabetes knowledge and health information-seeking behaviors among patients with diabetes in rural areas of China’s economically underdeveloped regions during COVID-19, as well as a lack of up-to-date evidence on glycemic control and the incidence of complications among rural patients with diabetes.

**Objectives:**

To investigate the prevalence of glycemic control and complications among patients with diabetes in rural areas, to explore the current status and correlation of diabetes knowledge level and health information-seeking behavior, and to analyze the factors affecting diabetes knowledge level.

**Methods:**

From January 2022 to July 2022, we conducted a screening on diabetic complications and a questionnaire survey among 2,178 patients with diabetes in 15 county hospitals in rural areas of Guangxi Zhuang Autonomous Region. The patients’ knowledge level and health information-seeking behavior were investigated. Spearman correlation analysis was used to assess the correlation between diabetes knowledge and health information-seeking behavior. Multiple linear regression analysis was used to test how demographic information and health information-seeking behavior influenced the level of diabetes knowledge.

**Results:**

Of 2,178 patients with diabetes in rural areas, 1,684 (77.32%) had poor glycemic control, and the prevalence of diabetic complications was estimated to be 72.13%. Patients with diabetes had poor diabetes knowledge and health information-seeking behavior, and there is a strong positive correlation between them. Diabetes knowledge level was influenced by *per capita* household disposable income, occupational status, gender, age, ethnicity, family history of diabetes, insulin use, glycated hemoglobin, education level, number of complications and health information-seeking behavior.

**Conclusion:**

Patients with diabetes in rural areas have poor glycemic control and a high incidence of diabetic complications. Patients with diabetes in rural areas have poor knowledge and inadequate health information-seeking behavior. Systematic and standardized education should be provided to improve patients’ diabetes knowledge and thus improve their self-management ability.

## 1 Introduction

Health information-seeking behaviors are active or purposeful actions taken by individuals to find information about their health, expressing the patient’s active pursuit of health and contributing to psychosocial adjustment (1). Effective information-seeking behavior aims to improve the supply of educational information and meet the needs of individuals for educational information (2). Providing education and information is an important component of chronic disease management strategies, and investigating health information-seeking behaviors at the individual level in the context of the diabetes pandemic is important for improving educational approaches.

The Guangxi Zhuang Autonomous Region is in the southern region of China, where the economy is underdeveloped, and the education level of people in rural areas is low. The rolling hills makes it difficult for patients with diabetes to access medical care in rural areas, and a large number of ethnic groups leads to complex living habits. People mostly eat a traditional diet high in carbohydrates, such as white rice, which can lead to an increased risk of diabetes and difficulty in its management. The lack of evidence on the level of diabetes knowledge and the current state of health information-seeking behaviors has hindered healthcare providers from targeting rural patients with diabetes for health management. The burden of diabetes in China is increasing, with the estimated prevalence of diabetes rising to 12.4% (3); diabetic nephropathy occurs in about 20–40% of patients with diabetes (4). In China, diabetes complications place a heavy economic and psychosocial burden on patients, but regional data on complications such as diabetic retinopathy and neuropathy are still lacking, and there is an even greater lack of up-to-date evidence on the prevalence of complications among rural patients with diabetes in economically underdeveloped areas (5). Guangxi Zhuang Autonomous Region is a region where Zhuang and many ethnic minorities gather, and studies have shown that the prevalence of diabetes in the Zhuang population is 12.0% (6). The survey by the Guangxi Center for Disease Control and Prevention shows that the degree of knowledge of diabetes among rural residents is lower than that of urban residents (7). The lack of evidence from existing studies during major public health events such as the COVID-19 epidemic can reflect the ability of patients with diabetes in rural areas to cope with epidemics and to care for themselves when coping with diabetes. This could reflect the self-care ability of patients with diabetes in rural areas when coping with epidemics. In the past, the health management of chronic diseases was mainly concentrated in urban hospitals. The health management for rural patients with diabetes was less, and the gap is still obvious. The China DIA-LEAD study shows that diabetes is still underdiagnosed and under-treated among patients (8); further efforts should be made to encourage patients to raise awareness of diabetes and its complications and to improve their health information-seeking skills to achieve good diabetes health management.

As the health needs of rural patients with diabetes increase, healthcare providers should improve the health management model of rural patients with diabetes to reduce the occurrence and development of complications (9, 10). During the COVID-19 period, with policy changes in rural areas to implement varying degrees of personnel travel control, the majority of patients with non-acute phases of chronic diseases are in a state of self-management at home (11), there are obstacles to receiving knowledge about diabetes management, the current state of research on diabetes knowledge levels and health access behaviors of patients with diabetes in rural areas during the COVID-19 period is insufficient, and the prevalence of diabetic complications lacks up-to-date evidence. Therefore, this study investigated the health management model of rural patients with diabetes in Guangxi. Therefore, this study investigated the prevalence characteristics, knowledge level, and health information-seeking behavior of diabetic complications in rural areas of Guangxi, and healthcare providers can provide health education and medical services to patients for early prevention and management to achieve precise prevention and treatment.

## 2 Background

Diabetic complications include macrovascular complications, microvascular complications, and diabetic foot, which result in increased mortality, renal failure, and decreased quality of life. Chinese Diabetes Prevention and Control Guidelines (4) state that the prevalence of diabetes in China is 11.2%, about 20–40% of patients will develop diabetic nephropathy, and the incidence of new foot ulcers within 1 year is 8.1% among patients with diabetes over 50 years of age. Timely recognition and control of the occurrence and development of diabetic complications are crucial for patients, but surveys show that Chinese patients with diabetes have a low diabetes awareness rate and poor diabetes knowledge (12).

Diabetes education plays an important role in diabetes management. Studies have shown that education can not only improve adherence to complication screening in patients with diabetes (13), but it can also reduce the occurrence of complications in patients and is cost-effective (14, 15). The Lancet Commission on Diabetes concluded that bringing expertise to benefit people with or at risk of diabetes can reduce unnecessary burdens on individuals, families, and society. Using data-driven, integrated, team-based care can reduce all-cause mortality in people with diabetes by 20–60% (16). The high prevalence of diabetes in rural China and the low rates of diabetes awareness, treatment, and control have become a pressing public health issue for diabetes prevention and management (17). Rural areas are relatively economically disadvantaged, healthcare awareness is weak, and there is a general lack of ability to seek information about diabetes among people with diabetes (18). In this situation, it is more important for medical institutions to improve the screening and management of diabetes and its complications, expand rural patients’ access to health information and provide health education to enhance the level of diabetes prevention and control.

In China, as the demand for quality healthcare resources for people with diabetes increases, the government has developed the “Health China 2030” plan, which calls for shared resources and collaboration among hospitals to jointly manage the health of the population (10). Therefore, this study investigated the prevalence of diabetes complications in rural areas of Guangxi and explored the knowledge level and health information-seeking behavior of patients with diabetes, which will provide international evidence to improve the knowledge level and expand the educational pathway of patients with diabetes in rural areas.

## 3 Methods

### 3.1 Aims

The objectives of this study were as follows: (1) to investigate the prevalence of glycemic control and complications among patients with diabetes in rural areas, (2) to investigate the current status and correlation of patients’ diabetes knowledge and health information-seeking behavior, and (3) to analyze the factors affecting the level of diabetes knowledge.

### 3.2 Design

This study is a multicenter cross-sectional study.

### 3.3 Participants

This study was completed from January 2022 to July 2022 to screen patients with diabetes for diabetic complications in the Guangxi Zhuang Autonomous Region, China. We used a multi-stage stratified sampling method to select a representative sample, with one city in each region randomly selected based on geographic region (central, eastern, southern, western, and northern) in the first stage and three counties randomly selected in the selected cities in the second stage, for a total of 15 counties selected for the survey. The inclusion criteria were as follows: (1) meeting the diabetes diagnosis criteria of the Chinese Diabetes Association in 2020 (4), (2) age ≥ 18 years, and (3) household registration and current residence in the surveyed area of rural. Exclusion criteria were as follows: (1) patients with gestational diabetes, (2) those with a history of dementia and other psychiatric disorders are known from medical information or after asking family members, and (3) the patient is in the acute phase of the disease and is unable to cooperate with the investigation. The protocol was approved by the Ethics Committee of the People’s Hospital of Guangxi Zhuang Autonomous Region. All administrators of the hospitals involved in this study gave informed consent, and all participants provided written informed consent.

### 3.4 Sample size

In this study, a total of 150 patients with diabetes in rural areas were included by conducting a pretest with 10 patients with diabetes in each of the 15 counties included, and the results showed that there were 116 patients suffering from diabetes complications, with a prevalence of 77.33%. According to the formula for calculating the sample content of multistage stratified sampling (19): *n* = *Z*^2^_1-*α*/2_ (*1-P*) *P/δ*^2^ × *deff* (where: *Z*_1-*α*/2_ = 1.96; *P* is the prevalence rate, which was 77.33% in this study; *deff* is the design effect (complex sampling efficiency), which was set to 2 in this study to take into account the differences in prevalence rates in different regions; and *δ* is the tolerance error, which was set to 0.1 in this study), the number of samples to be taken in each county was calculated as 136 cases. Considering the data inefficiency (possible missing data in the survey), the sample size was expanded by 10%, and it was expected that the number of people with diabetes needed to be surveyed in each county was 152, and a total of 2,280 cases of diabetes needed to be surveyed in the 15 counties.

### 3.5 Data collection

The study consisted of questionnaires, anthropometric measurements, and blood collection at local hospitals by trained investigators who measured height and weight using a mechanical anthropometer and a regularly calibrated electronic scale. The investigators also helped patients fill out questionnaires using standardized instructions. Investigators recorded glycosylated hemoglobin values and complication screening results for patients with diabetes at 15 hospitals, and the results were entered into Excel and sent to us *via* the Internet. Once the questionnaires were completed, they were sent to us via the Internet. We summarized the data from the 15 hospitals and saved them in an Excel spreadsheet. All data were independently verified by two researchers and entered into SPSS for statistical analysis, and all information was stored in a confidential computer to prevent disclosure.

### 3.6 Instruments

#### 3.6.1 Sociodemographic information

Individual socio-demographic factors, including gender, ethnicity, and whether they were taking diabetes medication and insulin, were investigated. In addition, patients were asked about their *per capita* household disposable income [Classified according to the 2021 income standards for rural residents surveyed by the China Statistics Bureau in CNY 18931/USD 2631 (20)].

#### 3.6.2 Screening for complications of diabetes and glycosylated hemoglobin

Based on the Chinese Diabetes Association’s Diabetes Diagnostic Criteria 2020 (4). The diagnostic criteria and screening process for diabetic complications in the 2020 Chinese Diabetes Association Diagnostic Criteria for Diabetes Mellitus were conducted by a collaboration of physicians and nurses from 15 hospitals to screen patients with diabetes for complications, including diabetic nephropathy, diabetic retinopathy, diabetic neuropathy, diabetic lower extremity arteriopathy, and diabetic foot disease. Venous blood was drawn from subjects by nurses to measure their glycosylated hemoglobin, which was reported to us, and HbA1c was reported in %.

#### 3.6.3 The audit of diabetes′ knowledge

The Audit of Diabetes′Knowledge (ADKnowl) was developed by Speight and Bradley (21) developed in 2001 and revised in 2003. The Chinese version of the scale was translated and revised by Weiyan (22), and the content validity index, retest reliability and internal consistency Cronbach’s alpha were 0.923, 0.997, and 0.909. The questionnaire consists of a 26-item set (111 items) reflecting the level of knowledge of patients with diabetes on eight dimensions related to treatment, when sick, hypoglycemia, effects of physical activity, reducing the risk of complications, effects of smoking/alcohol, foot care, and diet and food. The scale included three options: “correct,” “incorrect,” and “do not know.” One mark for the correct answer, no mark for the wrong answer or does not know.

#### 3.6.4 Health information-seeking behavior

The Health Information-Seeking Behavior Scale was developed by Kalantzi et al. (23) developed, and the Chinese version of the scale was translated and revised by Meifeng (24). The content validity index of the Chinese version of the scale was 0.920, the retest reliability was 0.882, and the overall Cronbach’s alpha of the scale was 0.866. It reflects eight dimensions of diabetes health information needs: medical personnel pathway, book pathway, Internet pathway, interpersonal communication pathway, subjective factors of information barriers, objective factors of information barriers, and cultural level of information barriers, with a total of 38 items. The scale was scored on a 5-point Likert scale, with higher scores indicating more significant health information-seeking behaviors of patients.

### 3.7 Ethical considerations

The authors’ hospital received approval from the ethics committee (approval number: KY-KJT-2021-26); the questionnaire described the voluntary and confidential nature of participation, and all hospital administrators and participants gave informed consent.

### 3.8 Data analysis

Data were imported and analyzed using IBM SPSS 26.0 (SPSS Inc.). Before importing the data for analysis, we excluded data with incomplete information (height and weight of patients could not be obtained accurately due to physical factors *N* = 8, refusal to undergo examination for complications *N* = 27, refusal to complete the questionnaire *N* = 39, incomplete questionnaire *N* = 28, total *N* = 102). Categorical variables such as ethnicity, gender, and prevalence of diabetes complications in the general data were described using composition ratios. The normality of continuous type variables such as age and duration of diabetes mellitus was checked before analysis, and normally distributed data were expressed as mean ± standard deviation, and comparisons between groups were made using the two independent-samples *t*-test; non-normally distributed data were expressed as median (interquartile range), and comparisons between groups were made using non-parametric tests. Continuous variables were analyzed for correlation with ADKnowl questionnaire scores using *Pearson* correlation (where both sets of variables conformed to a normal distribution) or *Spearman* correlation (where at least one set of variables did not conform to a normal distribution); dichotomous variables using non-parametric tests; stratified and categorical data were analyzed using analysis of variance (*ANOVA*), and non-parametric tests were used when variances were unequal. The effect of demographic information on diabetes knowledge was assessed for differences using a one-way ANOVA. Factors that differed in the one-way ANOVA were included in multiple linear regression, which was used to analyze how demographic information and health information-seeking behavior affected diabetes knowledge levels, and the variable assignments in the multiple linear regression are shown in Table 1. *p* < 0.05 was considered statistically significant.

**Table 1:** Assigning values to variables.

Variables	Assigning values
Gender	Female = 0; Male = 1
Family history of diabetes	No = 0; Yes = 1
Use diabetes drug	No = 0; Yes = 1
Use insulin	No = 0; Yes = 1
Educational level	Illiterate = 0; Elementary school degree = 1; Junior high school degree = 2; Senior high school degree = 3; Associate degree = 4; Bachelor degree and above = 5
Number of complications in patients with diabetes	None = 0, One = 1, Two = 2, Three = 3; Four = 4; Five = 5
Household disposable income *per capita*	<18,931 CNY (2631USD) = 0; ≥18,931 CNY (2631USD) = 1
Occupational status	Not employed = 0, Employed = 1
Ethnicity	Han (Z_1_ = 1, Z_2_ = 0); Zhuang (Z_1_ = 0, Z_2_ = 1); Other (Z_1_ = 0, Z_2_ = 0)

### 3.9 Validity, reliability, and rigor

Strict quality assurance and control were implemented in this study, and all investigators underwent standardized training in data collection to familiarize them with the purpose of the study and the method of collection for each indicator. A carefully selected, validated questionnaire was administered to collect the data. The administrators of the 15 hospitals were informed of the purpose of the study when contacted, explaining the methods of screening for diabetic complications, the content of the questionnaire, and instructions for completing it to ensure the validity and consistency of the data across hospitals. After the information on patients with diabetes was collected from each hospital, we checked and organized the data and verified the abnormal values with the investigators to ensure the validity and reliability of the data.

## 4 Results

### 4.1 Respondent characteristics

General data of 2,178 patients with diabetes from rural areas were statistically analyzed. The mean age of the participants was 63.25 (*SD* 12.71; range: 19–99 years), of which 1,204 participants were male (55.28%), most commonly with Junior high school education (*N* = 684; 31.40%). The mean duration of diabetes among participants was 7.96 (*SD* 4.07; range: 1–24 years), with the majority having type 2 diabetes (*N* = 2,123; 97.47%). There were 1,571 patients (72.13%) with diabetic complications. The demographic characteristics of the participants are shown in Table 2.

**Table 2:** Sample characteristics and univariate analysis.

Variables		Mean (SD)	*N* (%)	Score of diabetes knowledge	Statistics	*p*
Gender	Female		974 (44.72)	48 (45, 52)	−13.590^a^	<0.001
	Male		1,204 (55.28)	46 (43, 49)		
Age		63.25 (12.71)		47 (44, 50)	0.183^c^	<0.001
Ethnicity	Han		1,271 (58.36)	47 (44, 50)	21.236^b^	<0.001
	Zhuang		763 (35.03)	47 (44, 50)		
	Other		144 (6.61)	45 (42, 48)		
Marital status	Married		2020 (92.7)	47 (44, 50)	−0.438^a^	0.661
	Unmarried		158 (7.3)	46.5 (44, 50)		
Educational level	Illiterate		256 (11.75)	44 (40, 47)	82.852^b^	<0.001
	Elementary school degree		504 (23.14)	46 (43, 48)		
	Junior high school degree		684 (31.40)	47 (44, 50)		
	Senior high school degree		507 (23.28)	49 (45.5, 52)		
	Associate degree		180 (8.26)	50 (47, 53)		
	Bachelor degree and above		47 (2.17)	52 (48, 54)		
Occupational status	Employed		177 (8.1)	50 (47, 53)	−8.963^a^	<0.001
	Not employed		2001 (91.9)	47 (44, 50)		
BMI		23.06 (3.16)		47 (44, 50)	0.006^c^	0.767
Smoking	Yes		396 (18.18)	47 (44, 50)	−0.555^a^	0.579
	No		1782 (81.82)	47 (44, 50)		
Drinking	Yes		496 (22.77)	47 (44, 50)	−0.752^a^	0.452
	No		1,682 (77.23)	47 (44, 50)		
Have medical insurance	Yes		2,153 (98.9)	47 (44, 50)	−0.564^a^	0.572
	No		25 (1.1)	48 (44, 51)		
Household disposable income *per capita*	_≥_18,931 CNY (2631USD)		822 (37.7)	49 (46, 52)	−15.667^a^	<0.001
	_<_18,931 CNY (2631USD)		1,356 (62.3)	46 (43, 49)		
Diabetes type	Type 2 diabetes		2,123 (97.47)	47 (44, 50)	0.815^a^	0.665
	Type 1 diabetes		43 (1.97)	48 (42.5, 51)		
	Special type of diabetes		12 (0.56)	48 (45.5, 50.5)		
Diabetic duration (years)		7.96 (4.07)		47 (44, 50)	−0.013^c^	0.558
Family history of diabetes	Yes		317 (14.55)	50 (47, 53)	−12.737^a^	<0.001
	No		1861 (85.45)	46 (43, 49)		
Use diabetes drug	Yes		1,349 (61.94)	47 (44, 51)	−7.182^a^	<0.001
	No		829 (38.06)	46 (43, 49)		
Use insulin	Yes		913 (41.92)	49 (46, 52)	−16.723^a^	<0.001
	No		1,265 (58.08)	45 (43, 48)		
Glycated hemoglobin		8.60 (2.18)		47 (44, 50)	−0.515^c^	<0.001
Prevalence of diabetes complications	Diabetic Nephropathy		508 (23.32)			
	Diabetic Retinopathy		487 (22.36)			
	Diabetic neuropathy		635 (29.16)			
	Diabetic lower extremity arteriopathy		535 (24.56)			
	Diabetic foot		214 (9.83)			
Number of complications in patients with diabetes	0		607 (27.87)	49 (46, 52)	83.117^b^	<0.001
	1		895 (41.09)	47 (44, 50)		
	2		554 (25.44)	45 (42, 48)		
	3		112 (5.14)	43 (41, 46)		
	4		10 (0.46)	41.5 (39, 47)		
	5		0 (0)	0 (0)		
Score of health information-seeking behavior		109.45 (18.50)		47 (44, 50)	0.529^c^	<0.001

a: non-parametric test, b: ANOVA, c: Spearman correlation test.

### 4.2 Glycemic control and prevalence of complications in patients with diabetes

The mean value of glycosylated hemoglobin in patients with diabetes in rural areas of Guangxi Zhuang Autonomous Region was 8.60 (*SD* 2.18), in which 77.32% (*n* = 1,684) of patients with diabetes had poor glycemic control (≥7.0%). The prevalence of diabetic nephropathy, diabetic retinopathy, diabetic neuropathy, diabetic lower limb arteriopathy, and diabetic foot disease was 23.32, 22.36, 29.16, 24.56, and 9.83%, respectively (Table 2).

### 4.3 Knowledge level of patients with diabetes

The mean score for the level of knowledge about diabetes among people with diabetes in rural areas was 46.97 (*SD* 4.72; range: 29–62). The mean scores for diet and food, diabetes treatment, when sick, foot care, the effect of physical activity, the effect of smoking/alcohol, risk of complications reduction, and hypoglycemia were 8.83 (*SD* 2.24), 6.08 (*SD* 2.99), 3.53 (*SD* 1.31), 9.57 (*SD* 2.16), 3.82 (*SD* 1.31), 4.65 (*SD* 1.12), 5.04 (*SD* 1.95), and 5.44 (*SD* 1.86) (Table 3). The highest-scoring item (toenails should not be trimmed to a pointed shape) and the lowest-scoring item (foot problems should not be safely treated by yourself) were correct at 77.82 and 9.41%, respectively.

**Table 3:** Level of diabetes knowledge and health information-seeking behavior.

		Mean	SD	Range	Min	Max
Diabetes knowledge	Total score	46.97	4.72	33	29	62
	Diet and food	8.83	2.24	11	4	15
	Treatment	6.08	2.99	15	0	15
	When ill	3.53	1.31	7	0	7
	Foot care	9.57	2.16	11	5	16
	Effects of physical activity	3.82	1.31	8	0	8
	Effects of smoking/alcohol	4.65	1.12	8	0	8
	Reducing the risk of complications	5.04	1.95	11	0	11
	Hypoglycemia	5.44	1.86	11	0	11
Health information-seeking behavior	Total score	109.45	18.50	99	54	153
	Diabetes health information needs	34.35	6.44	43	14	57
	Medical personnel pathway	18.92	4.43	18	7	25
	Book pathway	10.46	3.27	14	5	19
	Internet pathway	4.44	1.61	7	2	9
	Interpersonal communication pathway	12.63	3.10	14	4	18
	Subjective factors of information barriers	8.62	1.96	9	4	13
	Objective factors of information barriers	8.75	2.24	10	3	13
	Cultural level of information barriers	11.27	2.84	13	5	18

### 4.4 Health information-seeking behavior of patients with diabetes

The mean score of health information-seeking behavior of patients with diabetes in rural areas was 109.45 (*SD* 18.50; range: 54–153). The mean scores for the eight dimensions of diabetes health information needs, medical personnel route, book route, Internet route, interpersonal communication route, subjective factors of information barriers, objective factors of information barriers, and literacy level of information barriers were 34.35 (*SD* 6.44), 18.92 (*SD* 4.43), 10.46 (*SD* 3.27), 4.44 (*SD* 1.61), 12.63 (*SD* 3.10), 8.62 (*SD* 1.96), 8.75 (*SD* 2.24), and 11.27 (*SD* 2.84) (Table 3). The most important health information-seeking route for patients with diabetes was a physician (*Mean* 3.33, *SD* 1.01) and the most important barrier factor for health information-seeking behavior was cost (*Mean* 2.98, *SD* 0.87).

### 4.5 Factors influencing the level of knowledge of patients with diabetes

There was a strong positive correlation between diabetes knowledge level and health information-seeking behavior (*r* = 0.529, *p* < 0.001) (Table 2). Multiple regression analysis showed that older patients with diabetes (*β* = 0.144, *p* < 0.001) and patients with diabetes with higher education levels had higher knowledge levels (*β* = 0.159, *p* < 0.001), and disposable income *per capita* exceeded the income standard of rural residents (*β* = 0.111, *p* < 0.001) and a higher level of knowledge among people with diabetes with good health information-seeking behavior (*β* = 0.176, *p* < 0.001). Knowledge was poorer in patients with diabetes with more complications (*β* = −0.253, *p* < 0.001), women (*β* = −0.186, *p* < 0.001), and those with lower glycated hemoglobin levels (*β* = −0.283, *p* < 0.001) had better knowledge. Finally, patients with diabetes with occupation (*β* = 0.134, *p* < 0.001), with family history of diabetes (*β* = 0.150, *p* < 0.001) and using insulin (*β* = 0.166, *p* < 0.001). Patients had a better level of knowledge (Table 4).

**Table 4:** Multiple regression analysis showing factors influencing diabetes knowledge level.

Variables	B	SE	β	t	p
Constants	43.131	0.701	–	61.509	<0.001
Gender	−1.768	0.123	−0.186	−14.418	<0.001
Age	0.054	0.005	0.144	10.839	<0.001
Family history of diabetes	2.011	0.178	0.150	11.298	<0.001
Use diabetes drug	−0.050	0.129	−0.005	−0.384	0.701
Use insulin	1.586	0.128	0.166	12.348	<0.001
Glycated hemoglobin	−0.612	0.035	−0.283	−17.558	<0.001
Educational level	0.620	0.055	0.159	11.292	<0.001
Number of complications in patients with diabetes	−1.358	0.069	−0.253	−19.591	<0.001
Household disposable income *per capita*	1.084	0.135	0.111	8.007	<0.001
Occupational status	2.307	0.221	0.134	10.432	<0.001
Score of health information-seeking behavior	0.045	0.004	0.176	10.395	<0.001
Ethnicity: Other	0				
Han	0.640	0.249	0.067	2.571	0.010
Zhuang	0.359	0.257	0.036	1.395	0.163

Adjusted R^2^ = 0.648, F = 308.622, *p* < 0.001.

## 5 Discussion

The level of glycemic control in patients with diabetes is poorer in rural areas. In developing countries, glycemic control in patients with type 2 diabetes has not been optimal over the past 12 years (25). China, as the largest developing country in the world, still needs to improve the level of glycemic control among patients with diabetes. The present study showed that the poorer glycemic control of patients with diabetes in rural areas of Guangxi may be related to the poorer access to medical resources for rural populations located in less economically developed areas. This finding is similar to a study in northern Jordan, where 92.7% of participants had poor glycemic control (HbA1c ≥ 7%), and the mean value of HbA1c was 9.29% (26). The study showed that glycemic control was significantly associated with short-term prognosis and long-term respiratory sequelae in patients with COVID-19 combined with T2D and that good glycemic management had a positive impact on the prognosis of COVID-19 (27). Adults with type 2 diabetes who develop the disease at a young age are less likely to achieve glycemic control 1 year after diagnosis, suggesting that tailored care is needed to improve the prognosis of this group (28). The above findings suggest that healthcare professionals need to better organize diabetes care to improve self-management and achieve optimal glycemic control. Influenced by the lack of transportation in rural areas and epidemics such as COVID-19 that result in varying degrees of travel control, people with diabetes in rural areas are at a disadvantage in their ability to cope with changes in the disease, healthcare professionals should provide health education and medical care to people with diabetes in rural areas based on their level of knowledge and educational needs, and administrations should promulgate policies that address how to preserve the health of people with diabetes in rural areas during an epidemic.

The prevalence of diabetes complications is higher in rural areas. In China, changes in lifestyle and diet may lead to obesity and reduced physical activity as economic development and urbanization accelerate. Studies have shown that low socioeconomic status is associated with poor metabolic control and a higher prevalence of diabetes complications and that patients with diabetes with low education have the highest odds of retinopathy, cardiovascular disease, and cerebrovascular disease (29). HbA1c is associated with the risk of complications, and once hyperglycemia occurs, people with all forms of diabetes are at risk for the same chronic complications, although the rate of progression may vary. A meta-analysis of the Chinese population showed that patients with diabetes living in rural areas were more likely to develop diabetic retinopathy than those in urban areas, possibly due to the lower level of economic development in rural China, where limited primary health resources lead to delayed diagnosis of diabetes and non-optimal management of diabetes (awareness, treatment and control of diabetes were lower in rural residents than in urban residents) (30). Due to an aging population, the prevalence of chronic diseases increases with age, multimorbidity affects the quality of patient survival, and metabolic diseases are the most important diseases in almost all age groups, suggesting that a wider age range requires appropriate guidelines and a flexible care management support system to benefit a larger number of patients (31). The prevalence of diabetes complications highlights the importance of regular surveillance and the need for healthcare providers to plan future health services appropriately to achieve early detection and comprehensive treatment of diabetes complications.

The level of diabetes knowledge among patients with diabetes in rural areas is poor. In this study, patients with diabetes had a better awareness that their toenails should not be trimmed to a pointed shape and a poorer awareness that foot problems could not be safely treated by themselves. Knowledge plays an important role in the self-management of diabetes; the risk of diabetes complications is influenced by the patient’s level of knowledge, and the provision of educational services and time spent with patients with diabetes is highly correlated with better practice and disease outcomes (32). Studies have shown that improving patients’ knowledge through enhanced diabetes education and self-management skills and giving them positive attitudes toward achieving and maintaining good glycemic control can be effective in achieving glycemic control (33). There are many ethnic minorities in the rural areas of Guangxi, and there are differences in the customs and living habits of different ethnic groups. Studies have shown that in the special cultural context, as the level of knowledge about diabetes improves, patients with diabetes’ glycemic control during Ramadan fasting becomes better (34). Studies have shown that lack of knowledge about diabetes is a major factor in delaying the diagnosis and treatment of patients with diabetes in rural areas of China (35) and that healthcare professionals should provide targeted screening, early intervention, and health education to patients to raise awareness of diabetes among rural diabetes patients and to encourage them to go to the hospital to receive healthcare services on a regular basis. Participants are more likely to engage in self-care if health professionals provide information in culturally appropriate and easy-to-understand language (36). This evidence provides a reference for the rural areas of Guangxi with complex cultural backgrounds and a concentration of ethnic minorities, suggesting that health professionals should improve their diabetes knowledge according to their lifestyle habits.

Poor health information-seeking behavior among people with diabetes in rural areas. The lower percentage of patients with diabetes in rural areas of Guangxi who acquired diabetes knowledge through the Internet may be related to poorer economic conditions, higher age, and lower education levels. Internet education suffers from the barriers of lack of Internet access and unstable connectivity, poor patient health literacy leading to an inability to accurately identify and match their own needs and health information resources, and inconsistency between Internet dietary management information and hospital treatments leading to the fear of unprofessional sources of information for patients’ knowledge of the Internet (37), which suggests that Internet education, although it can disseminate superior medical resources, needs improved methods to be applied in rural areas. Information-seeking ability declines with age, and for people with diabetes in rural China, the most common source of information is the physician, which is caused by the presence of one or two village doctors in each village or every few villages and the lack of matching nurses (18). Compared to physicians, nurses provide health education for longer periods, return more frequently, can improve patient satisfaction and quality of life, and may lead to better health outcomes for patients (38). The educational pathways are varied, and after receiving education from a dietitian, patients not only gain knowledge but also have a significantly lower glycemic index, suggesting that in future, management could improve glycemic control in rural patients with diabetes by training professional dietitians to provide services to them (39). Improving the ability of rural residents to seek information should be incorporated into diabetes management, and healthcare providers should provide patients with effective health education tools according to the actual situation, thus improving the knowledge of patients with diabetes.

The diabetes knowledge level is influenced by household disposable income *per capita*, occupational status, gender, age, ethnicity, family history of diabetes, insulin use, glycated hemoglobin, education level, number of complications, and health information-seeking behavior. The more extensive the source of health information, the easier it was for patients to gain knowledge about diabetes, and the better health information-seeking behavior, the higher the level of knowledge of patients. This finding is consistent with Katherine’s study (40). As the number of diabetes complications increased, the level of knowledge of patients with diabetes decreased, implying that the higher the level of diabetes knowledge, the easier it was for patients to achieve better glycemic control and thus to delay or control the onset and progression of complications (32, 33). The lower the level of education, the poorer knowledge level of patients, probably because lower literacy rates hinder health engagement behaviors throughout the life cycle, which leads to poorer health literacy and health promotion behaviors (41). The higher knowledge level of patients with a family history of diabetes may be because patients have regular contact with people with diabetes in their daily lives and have access to some of the knowledge and experience in providing healthcare for diabetes. As the *per capita* disposable household income increased, the knowledge level of patients with diabetes was better, probably because the higher the income, the better the self-care behavior and health literacy of the patients, leading to a higher knowledge level (42). It is an interesting finding that women have better knowledge of diabetes than men, suggesting that women adopt more self-protective behaviors to maintain their health when they are at risk for the disease (43). These findings can help medical professionals understand the level of diabetes knowledge among patients with diabetes based on the influencing factors and then select appropriate educational pathways to improve the knowledge of patients with diabetes based on their health information-seeking behavioral characteristics.

## 6 Limitations

This was a multicenter cross-sectional study in which we screened rural patients with diabetes for complications and administered questionnaires at 15 county hospitals. Although we selected rural patients with diabetes according to national diagnostic criteria, the models of diabetes complication screening equipment and glycosylated hemoglobin measurement equipment were not exactly the same in the 15 county hospitals, so the test results varied slightly from hospital to hospital. Screening equipment for diabetic complications is usually only available in hospitals at the county level and above because they are expensive, bulky and immobile. We were unable to move these devices to rural areas to conduct the study, so we chose rural patients with diabetes who came to the hospital for care, which should be adopted with caution when promoting the application.

## 7 Conclusion

Patients with diabetes in rural areas have poor glycemic control and a high incidence of diabetic complications. Patients with diabetes in rural areas have poor knowledge and inadequate health information-seeking behavior. Systematic and standardized education should be provided to improve patients’ diabetes knowledge and thus improve their self-management ability.

## Data availability statement

The data analyzed in this study is subject to the following licenses/restrictions: the datasets generated during and/or analyzed during the current study are available from the first author or corresponding author upon reasonable request. Requests to access these datasets should be directed to YW, 15163566100@163.com.

## Ethics statement

The studies involving humans were approved by Ethics Committee of People's Hospital of Guangxi Zhuang Autonomous Region. The studies were conducted in accordance with the local legislation and institutional requirements. The participants provided their written informed consent to participate in this study.

## Author contributions

YW: Writing – original draft. YZ: Writing – review & editing. TG: Writing – review & editing. JH: Writing – review & editing. GF: Writing – review & editing.
